# Hydrology, biogeochemistry and metabolism in a semi-arid mediterranean coastal wetland ecosystem

**DOI:** 10.1038/s41598-022-12936-5

**Published:** 2022-06-07

**Authors:** Béchir Béjaoui, Leila Basti, Donata Melaku Canu, Wafa Feki-Sahnoun, Hatem Salem, Sana Dahmani, Sabrine Sahbani, Sihem Benabdallah, Reginald Blake, Hamidreza Norouzi, Cosimo Solidoro

**Affiliations:** 1 National Institute of Marine Sciences and Technology, University of Carthage, 28 rue du 2 Mars 1934 Carthage Salammbô, Tunis, Tunisia; 2 Faculty of Marine Resources and Environment, Tokyo University of Marine Science and Technology, Tokyo, Minato 108-8477 Japan; 3 National Institute of Oceanography and Applied Geophysics, OGS, Borgo Grotta Gigante, 42/c, 34010 Sgonico, Trieste, Italy; 4 Laboratory of Hydraulics and Environment, National Engineering School of Tunis, University of Tunis, BP 37, 1002 Tunis, Tunisia; 5 University of Applied Sciences for Engineering and Economics, Treskowallee 8, 10318 Berlin, Germany; 6 National Institute of Agronomy of Tunisia, 43 Av. Charles Nicolle, 1082 Tunis, Tunisia; 7 Centre for Water Research and Technologies, Technople Borj Cedria, BP 273-8020, Tunis, Tunisia; 8 The City University of New York, New York City College of Technology, 300 Jay St, Brooklyn, NY 11201 USA; 9 International Centre for Theoretical Physics, ICTP, Strada Costiera, Trieste, Italy

**Keywords:** Ecology, Environmental sciences, Ocean sciences

## Abstract

A LOICZ Budget Model is applied to the Ichkeul Lake, a wetland ecosystem of the South Mediterranean-North African region, to evaluate its functioning in order to boost water management. The Ichkeul Lake water and nutrient budget, net ecosystem metabolism (NEM), nutrient availability, and their seasonal changes are estimated using field data. A considerable anthropogenic-driven amount of nitrogen is transferred into N_2_/N_2_O to the atmosphere during the dry season with predominance of denitrification-anammox processes. The primary production is impacted by forcing the ecosystem respiration to reduce the NEM so that the system is functioning as heterotrophic. Climate change and anthropogenic pressures are expected to exacerbate the current trends of water quality degradation, with possible negative impacts on Palearctic birds’ population. Mitigation actions are possible, through the implementation of National Wetland Management Strategies that include nutrient load and water resources management.

## Introduction

Wetlands are important wildlife ecosystems with abundant habitats and high productivity that play distinctive and unique roles on a planetary scale when it comes to climate regulation, biodiversity, food security, energy, blue carbon, water management, disaster risk reduction, human health, sustainable livelihood, and urban future^1–6^. Yet, wetlands are considered one of the most threatened habitats due to their vulnerability and attractiveness for human development^7^. Despite recent sustainable management attempts, most wetlands have undergone major disruptions, and the favorable areas of their well-conserved habitats are still declining^8^. In addition to habitat loss and degradation, the essential pressures are exerted by reduced watershed inputs due to the damming of rivers and the deterioration of water quality associated with an excess of nutrient inputs and organic matter from urbanization, industrial effluents, tourism, agriculture, land reclamation, and pollution as well as the overexploitation of biotic and abiotic resources under increasing climate change pressures^9–12^. These pressures cause changes in the hydrology, biogeochemistry and metabolism of wetland ecosystems disturbing their ecological status, biodiversity, and socio-economic services^12–14^.

Globally, up to 70% of wetlands have been lost since 1900 AD, including coastal wetlands that provide higher estimated values of services^15^. The latter, considered unique ecosystems at the land-sea continuum, are characterized by high spatial and temporal variability and provide the highest ecosystem services along with coral reefs, seagrass meadows, and algae beds^5,16,17^. In particular, coastal wetlands in arid and semi-arid regions, conspicuously in North Africa, are foci of human settlements and activities making them particularly vulnerable since their spatial range has been contracting and their ecological condition deteriorating^18,19^. Among these wetland ecosystems, the Ichkeul Lake is jeopardized by climatic and human pressures. This unique Lake is endowed with great national and international importance^20^. It has long been recognized as one of the most important preserved wetlands in the Mediterranean region and as an important overwintering site of Palearctic birds at a cross continental level, Africa-Europe^21–23^. The lake is part of a land-lake-lagoon-sea continuum and presents a rather spectacular hydrological functioning, with a seasonal reversal of the water exchange with the adjacent Bizerte Lagoon, which is in turn connected with the Mediterranean Sea. Thanks to its high ecological value and distinction of its hydrological functioning, the Ichkeul wetland is one of the few sites listed under three international conventions namely the Biosphere Reserves of 1977^24^, the World Heritage Convention of 1979^25^ and the Ramsar Convention of 1980^26^. It was also ratified as a national park in 1980. Since the 1980s, however, this area has been threatened by the construction of dams on the main rivers which drastically reduced the freshwater inputs to the Lake^23^. A sluice was also installed in 1993 at the outlet of the Lake at the Tinja channel to regulate the seawater fluxes between the Lake and the Lagoon of Bizerte. Nonetheless, these locks have caused the formation of new sedimentary deposits upstream of the channel^27^.

Anthropogenic and climate change pressures on coastal wetlands have often been related to excess algal production followed by significant drops in dissolved oxygen and/or the escape of Nitrogen from the systems in its gaseous forms as N_2_ and/or N_2_O as well as significant changes in Phosphorus budgets^28–31^. A budget approach developed in the Land–Ocean-Interactions in the Coastal Zone (LOICZ) project was applied to evaluate the net ecosystem metabolism under different seasonal conditions and to assess the system sensitivity to climate and anthropogenic pressures.

Despite their simplicity, budget models are very useful tools to understand and quantify the general features of the functioning of these systems and to point out the possible causes and solutions of dysfunctional events. In this context, the LOICZ model^28,40,41^ lends itself well the design of the general processes in a system such as the Lake Ichkeul. The LOICZ gives knowledge and understanding of the biogeochemical functioning of the system^42,43^, as a highly recommended model for coastal regions where the interactions between human activities and natural systems are very high^44,47^. In addition, LOICZ is used to test some schemes to assess the effects of nutrient loads into the system as well as the possible management intervention^42,43^.

The LOICZ biogeochemical model constitutes a dexterous tool to study the water and nutrient budgets of the Ichkeul wetland and provides nutrient fluxes as well as N and P metabolism in coastal waters^[34,35,42, ,43,48,49]^. The model uses field data of dissolved inorganic nitrogen (DIN) and dissolved inorganic phosphorus (DIP), calculates the net budget of DIN and DIP and compares them with the prepared for values, relying on classical stoichiometry, to calculate from that difference the Net Ecosystem Metabolism (NEM). This model has been applied to over 200 sites in the Americas, Central Asia, South-East Asia, the Black Sea, the Polar Regions, Oceania, the Northern Mediterranean, and sub-Saharan Africa ^52^. Recently, the application of LOICZ model to coastal systems with different flushing modes has settled a relation between water residence time and the NEM^43,49,50^. This approach is characterized by the simplicity of its application, its robustness, and its efficiency to evaluate the outcomes^48^.

Several studies have been carried out to evaluate the Ichkeul Lake ecological status^49^; however, quantitative studies based on numerical analyses are scarce. In particular, the numerical models as well as biogeochemical models commonly applied to assess the trophic status and water quality of coastal wetlands^12,50,51^ have never been applied to wetland ecosystems of the South Mediterranean-North African region. In this study, a LOICZ model and a Budget Model tool are applied for the first time to Ichkeul Lake to characterize its seasonal and spatial functioning and estimate the budgets of water and the key nutrients N and P, as well as their net retention/release in response to climate change and anthropogenic pressures. By dressing a full picture of the main functional features of the Lake, the present study aims to boost the management of a RAMSAR Coastal Wetland via improved water management strategies. This study is rooted in experimental observations carried out during a 5-month campaign.

## Results

### Physico-Chemical, Chemical and Chlorophyll a parameters

#### Temperature

**Figure 1:**
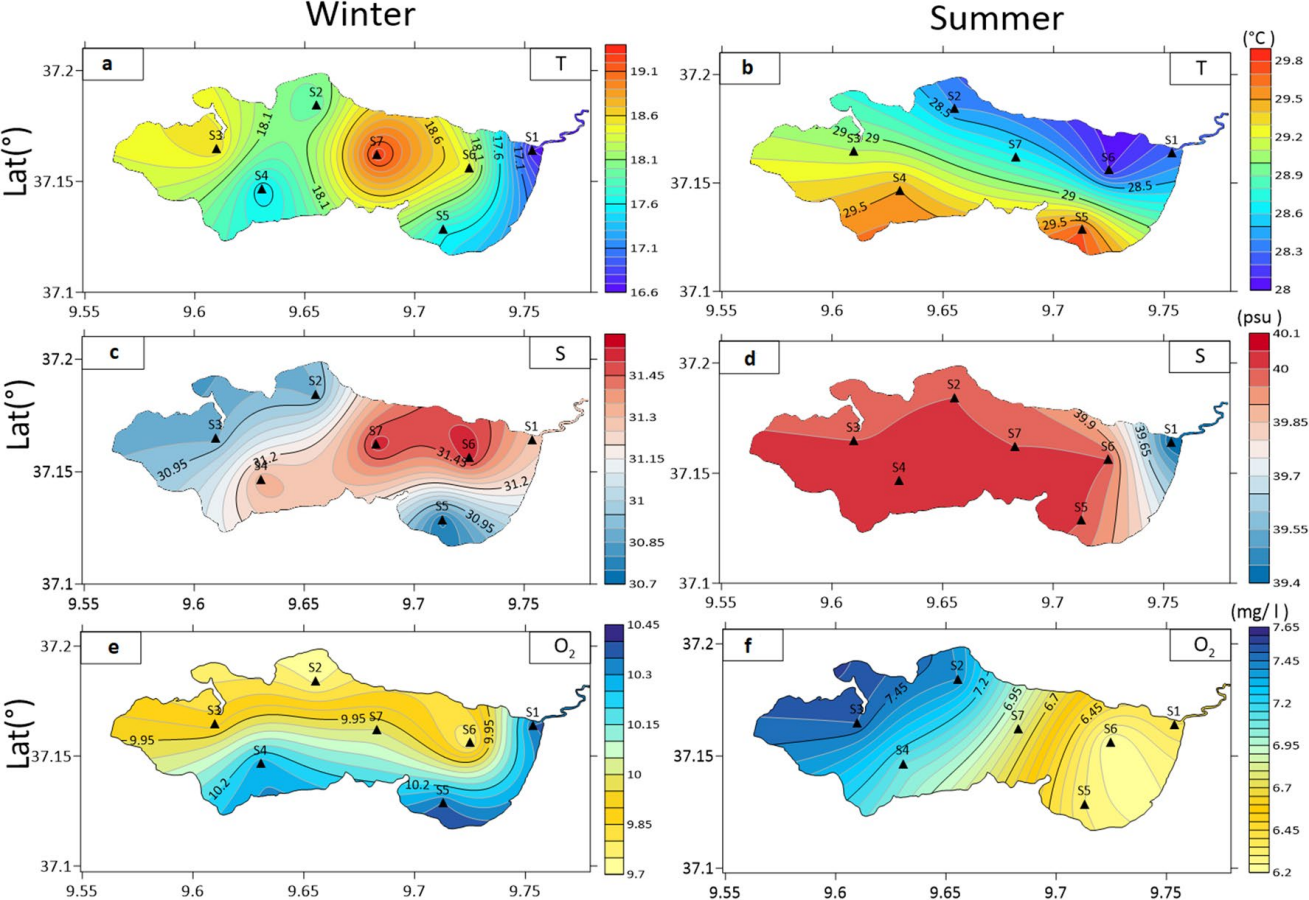
Spatial distribution of physico-chemical parameters in winter (*capital letter*) and summer (*apostrophic capital letter*) including Temperature (**a**, **b**), Salinity (**c**, **d**) and Dissolved Oxygen (**e**, **f**). Map created using Surfer 7.02.

In the Ichkeul Lake, due to its shallow depth, the temperature is tightly linked to that of the atmosphere. In the wet season, the water temperature is between 16.7 °C in S1 and 19.3 °C in S7 (Fig. 1a) where the water depth is relatively high. In the dry season, the Western and Southern sectors are characterized by higher water temperatures of about 25 °C due to water confinement and shallow depth (Fig. 1b).

#### Salinity

Salinity of the Ichkeul Lake was high throughout the study period between 2016 and 2017. The lowest value was about 30.7 recorded in winter then reached 40.0 in late summer (August). In winter and summer, the salinity was likely to be homogenously distributed with an average of 31.17 ± 0.3 (Fig. 1c) and 39.91 ± 0.2 (Fig. 1d), respectively. In winter, lower values were noticed at the outlet of the rivers carrying out freshwater. Meanwhile, in summer, they were recorded by the side of the Tinja channel where the lagoon salinity was about 38.6, lower than the other compartments of the lake.

#### Dissolved Oxygen

Similar to salinity, the DO was almost homogenous during the year with average values of 10 mg.l^−1^ ± 0.3 in winter (Fig. 1e) and 6.8 mg l^−1^ ± 0.5 in summer (Fig. 1f). In winter, the highest concentrations were in the South (S4 and S5) and Eastern sides (S1) of the lake, whereas, in summer, the highest concentrations were recorded in the Western side characterized by blooming of Phanerogams.

#### Turbidity

Relying on the overall field observations, the waters were turbid throughout the year, essentially due to the impact of meteorological conditions and also due to the morphology of the lake which is characterized by very shallow water depth. Seasonally, the waters were more turbid in winter during northwestern winds which force the sediment to re-suspend in the water column. However, in summer, the turbidity was relatively low in comparison to that observed in winter due to the moderate meteorological conditions. Spatially, the western sector was often distinguished by low turbidity due to the presence of phanerogams in addition to water confinement.

#### Dissolved phosphorus

**Figure 2:**
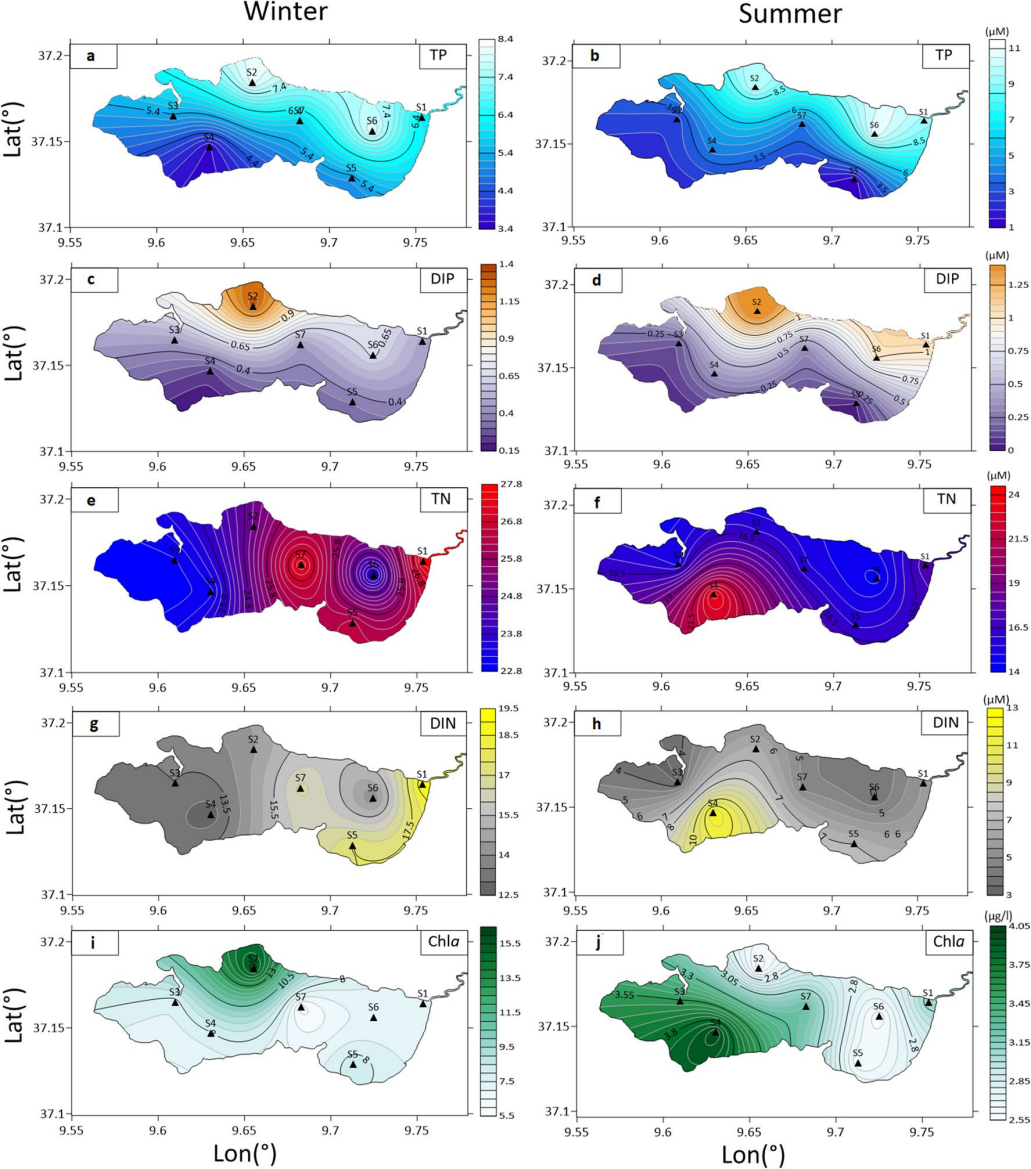
Spatial distribution of phosphorous, nitrogen components and Chlorophyll a in winter (*capital letter*) and summer (*apostrophic capital letter*), including Total Phosphorous TP (**a**, **b**), Dissolved Inorganic Phosphorus DIP (**c**, **d**), Total Nitrogen TN (**e**, **f**), Dissolved Inorganic Nitrogen DIN (**g**, **h**) and Chlorophyll a *Chla* (**i**, **j**). Map created using Surfer 7.02.

Overall, the Ichkeul Lake is characterized by low phosphorus levels during the sampling period. The distribution of the Total Phosphorus (TP) in the lake was almost homogeneous during the year with a high concentration observed in the northern sector of the lake and a lower value in the southern sector. In winter (Table 1), the total phosphorus varied between 3.6 µM in the southern sector where the Joumine River discharges and 8.1 µM in the Northern sector where the Douimis River discharges (Fig. 2a). In summer, we observed a similar distribution of the phosphorus in the lake as compared to winter (Fig. 2b), with an increase in values (Table 2) especially in the northern sector where the TP reached 10.9 µM.

**Table 1:** Physico-chemical and chemical parameters measured in Ichkeul Lake in winter (2017).

Station	T (°C)	S	O2 (mg l^−1^)	TN (µM)	DIN (µM)	TP (µM)	DIP (µM)	Chl*a* (µg l^−1^)
S1	16.7	31.3	10.4	27.5	18.9	6.1	0.5	7.0
S2	17.9	30.9	9.7	25.0	14.4	8.1	1.3	15.8
S3	18.6	30.9	9.9	23.0	13.5	5.4	0.5	7.7
S4	17.5	31.3	10.3	23.1	12.7	3.6	0.2	7.9
S5	17.7	30.7	10.4	26.5	17.5	5.3	0.4	8.4
S6	18.5	31.5	9.7	23.5	13.9	8.0	0.7	7.0
S7	19.3	31.5	10.0	27.4	16.7	5.9	0.5	5.8
Mean ± SD	18.0 ± 0.8	31.2 ± 0.3	10.1 ± 0.3	25.1 ± 2.0	15.4 ± 2.3	6.1 ± 1.6	0.6 ± 0.3	8.5 ± 3.3

T: Temperature, S: Salinity, O_*2*_: Dissolved Oxygen; TN: Total Nitrogen; DIN: Dissolved Inorganic Nitrogen; TP: Total Phosphorous; DIP: Dissolved Inorganic Phorsphorous; Chl*a*: Chlorophyll *a.*

**Table 2:** Physico-chemical and chemical parameters measured in Ichkeul Lake in summer (2016).

Station	T (°C)	S	O2 (mg l^−1^)	TN (µM)	DIN (µM)	TP (µM)	DIP (µM)	Chl*a* (µg l^−1^)
S1	28.3	39.4	6.4	17.5	5.6	9.3	1.1	3.1
S2	28.4	40.0	7.4	16.0	6.7	10.0	1.6	2.6
S3	29.1	40.0	7.5	15.4	3.5	2.7	0.2	3.6
S4	29.5	40.0	7.1	23.8	12.1	4.2	0.4	4.0
S5	29.7	40.0	6.3	15.9	7.0	1.2	0.1	2.6
S6	28.1	40.0	6.2	14.3	3.9	10.9	1.0	2.6
S7	28.7	40.0	6.8	15.7	4.8	4.0	0.3	3.3
Mean ± SD	28.8 ± 0.6	39.9 ± 0.2	6.8 ± 0.5	16.9 ± 3.2	6.2 ± 2.9	6.0 ± 3.9	0.7 ± 0.6	3.1 ± 0.6

T: Temperature, S: Salinity, O_*2*_: Dissolved Oxygen; TN: Total Nitrogen; DIN: Dissolved Inorganic Nitrogen; TP: Total Phosphorous; DIP: Dissolved Inorganic Phorsphorous; Chl*a*: Chlorophyll *a.*

The Dissolved Inorganic Phosphorus (DIP) varied between 0.2 µM and 1.3 µM in winter (Table 1), while it varied between 0.1 µM and 1.6 µM in summer (Table 2). The highest concentration is observed in the northern sector close to the Douimis River discharge in winter (Fig. 2c) as well as in summer (Fig. 2d), and the lowest values were recorded in the southern sector of the lake.

#### Dissolved nitrogen

The distribution of the Total Nitrogen (TN) in the lake is characterized by a non-uniform spatial distribution during the year. In winter (Table 1), a high concentration of about 27.5 µM was observed in the center of the lake and in the Tinja channel while the lowest value of 23.0 µM was observed in the western and in the North-eastern side (Fig. 2e). In summer (Table 2), a high concentration of about 23.8 µM was observed in the southern sector (Fig. 2f) where discharges of the Joumine River take place, whereas the lowest value of 14.3 µM was observed in the western sector and in the North-eastern side of the lake.

For the Dissolved Inorganic Nitrogen (DIN) (Fig. 2g) the level of concentrations is obviously higher in winter than in summer (Fig. 2h). In winter, the Eastern sector recorded the highest concentration (15.4 µM) while in summer the highest level was detected in the West-southern side (6.2 µM), loci for Joumine River discharge.

#### Chlorophyll *a*

The Chl*a* concentration revealed a clear contrast and variation between seasons. In winter, the average concentration reached 8.5 ± 3.3 μg.l^−1^ (Table 1), with the highest concentrations observed at S2 (Fig. 2i). In summer, the average concentration was relatively low, at about 3.1 ± 0.6 μg.l l^−1^ (Table 2), in the Western sector (S3, S4) (Fig. 2j).

### Multivariate statistical analysis

The PCA was performed for winter and summer separately to understand the correlation between the sampling area, Chl*a*, and chemical and physico-chemical parameters for each season, and also to highlight the seasonal functioning (Fig. 5).

**Figure 3:**
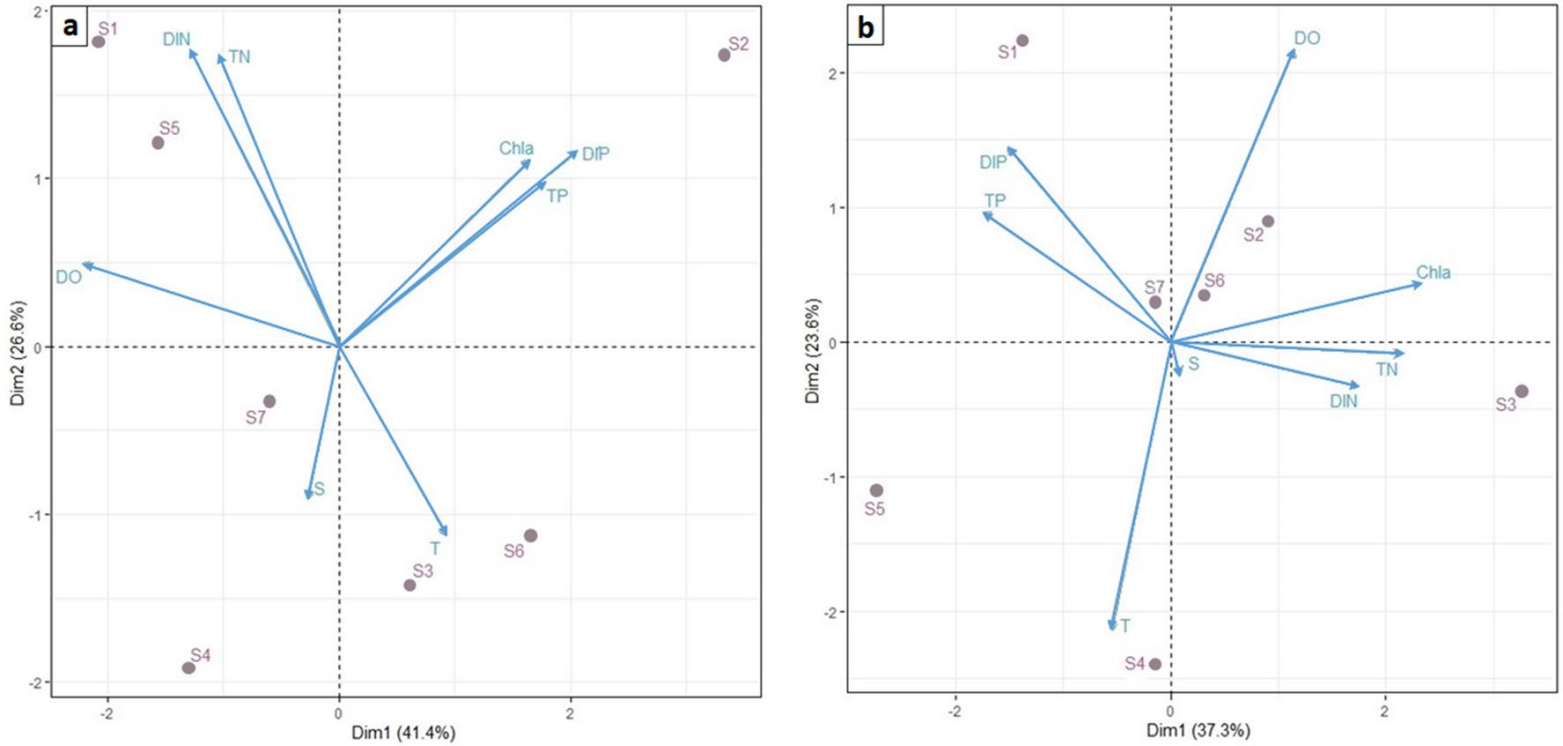
Principal Component Analysis (PCA). Plots of the PCA performed for Chlorophyll *a*, physical and chemical parameters according to winter (**a**) and summer (**b**) with sampling stations.

In winter (Fig. 3a), the first two components described 68% of the total variance. The first component (41.4%) tracked a combination of trophic related variables, phosphorus and Chlorophyll *a*, as opposed to dissolved oxygen, and discriminated the area close to the inlet (S1) and the coastal area localized close to the mouth of the Douimis River (S2). The second component (26.6%) mainly discriminated the nitrogen and the temperature between the area close to the inlet (S1) and the inner sector (S5) from one side and the southeast coast area (S3 and S4) which captured the impact of Sejnene and Melah Rivers.

In summer (Fig. 3b), when the lake received waters from the lagoon, the first two components accounted for 60.9% of the total variability. The first component (37.3% )—dominated by the combination of nitrogen, Chlorophyll *a*, and, with the opposite sign, phosphorous—discriminated the inner and western coastal regions close to the Douimis River (S3, high Chl*a*, high N, and low P) from the eastern stations close to Joumine and Tine Rivers (S5, high P, low N, and Chl*a*) and the inlet (S1, high P, low N, and Chl*a*).The second component (23,6%), however, described mainly gradients in temperature and dissolved oxygen between areas south and close to the Melah River (S4, high T, low DO) or Tine-Joumine system (S5) and northern stations close to the inlet of Tinja Channel (S1, low T, and high DO).

### LOICZ Results

#### Water and salt budget

**Figure 4:**
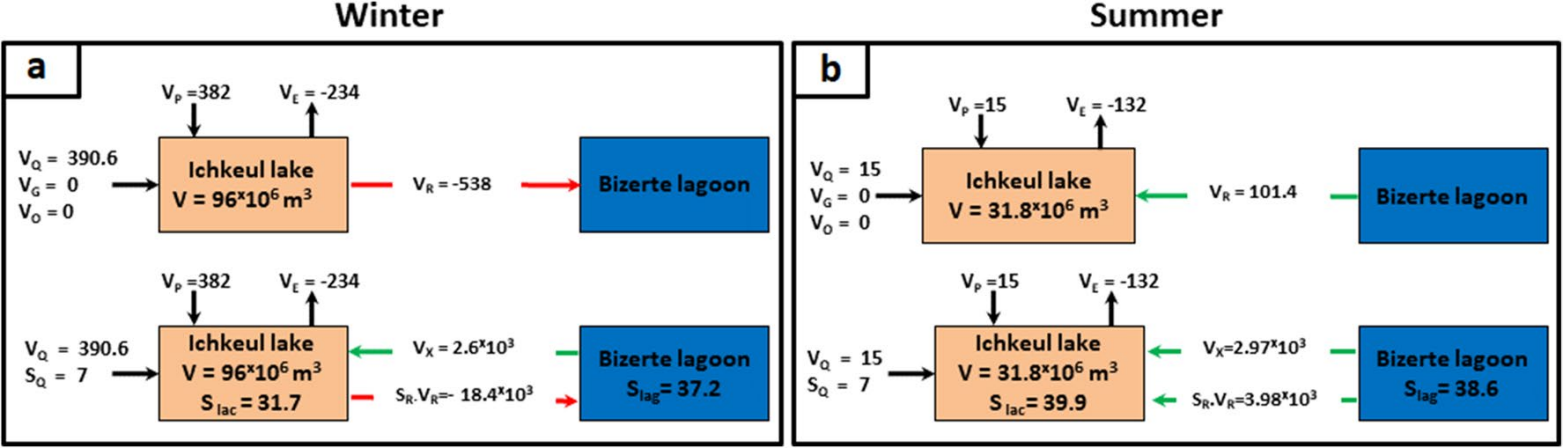
Generalized box diagrams for seasonal changes in hydrology and biogeochemistry.

During the winter season (Fig. 4a), the Ichkeul Lake received a quantity of freshwater from the six rivers (V_Q_ = 390.6 10^3^ m^3^.d^−1^) and a flux of direct precipitations (V_P_ = 381.7 10^3^ m^3^.d^−1^). Meanwhile, evaporation was about − 234.0 10^3^ m^3^.d^−1^. The water residual flow given as a result from the LOICZ model was equal to − 538.2 10^3^ m^3^.d^−1^. Its negative value indicates that water leaves the Ichkeul Lake towards the Bizerte Lagoon. At steady state, the V_R_ freshwater outflow carried salt by advection outside of the lake with the boundary salinity S_R_ being almost equal to 34.2. The advective salt delivery to the lagoon is given by the flux V_R_*S_R_ which was about − 18,406.4 10^3^ kg.d^−1^, thus explaining the lower salinity in the lake (31.7) than the lagoon (37.2). To remove the excess of salt in the latter, the water mixing flux V_X_ was computed so that V_X_ (S_lag_-S_lac_) was equal to -(V_R_S_R_ + V_Q*_S_Q*_). In this case, at steady-state, the mixing flux entering the lake from the lagoon was about 2,589.5 10^3^ m^3^.d^−1^. The residence time in winter was about 31 days.

In summer (Fig. 4b), the amount of freshwater flowing to the lake from precipitation (V_P_ = 15.1 10^3^ m^3^.d^−1^) and rivers (V_Q_ = 15.4 10^3^ m^3^.d^−1^) remained low compared to the losses by evaporation (V_E_ = -− 131.9 10^3^ m^3^.d^−1^) inducing lagoon waters to enter the lake (V_R_ = 101.4 10^3^ m^3^.d^−1^) thereby carrying salt to the lake which salinity increased to 40.0 while that of the lagoon was about 38.6. To equilibrate the slight salt difference between the two systems, the flux (V_R_S_R_ + V_Q*_S_Q*_) must be equal in opposite signs to V_x_ (S_lag_-S_lac_), and therefore V_x_ had to be equal to 3,097.2 10^3^ m^3^.d^−1^. In this situation, the residence time in the system was about 10 days, and the renewing water is mostly of lagoon origin.

#### Non-conservative materials budgets

##### DIP budget

**Figure 5:**
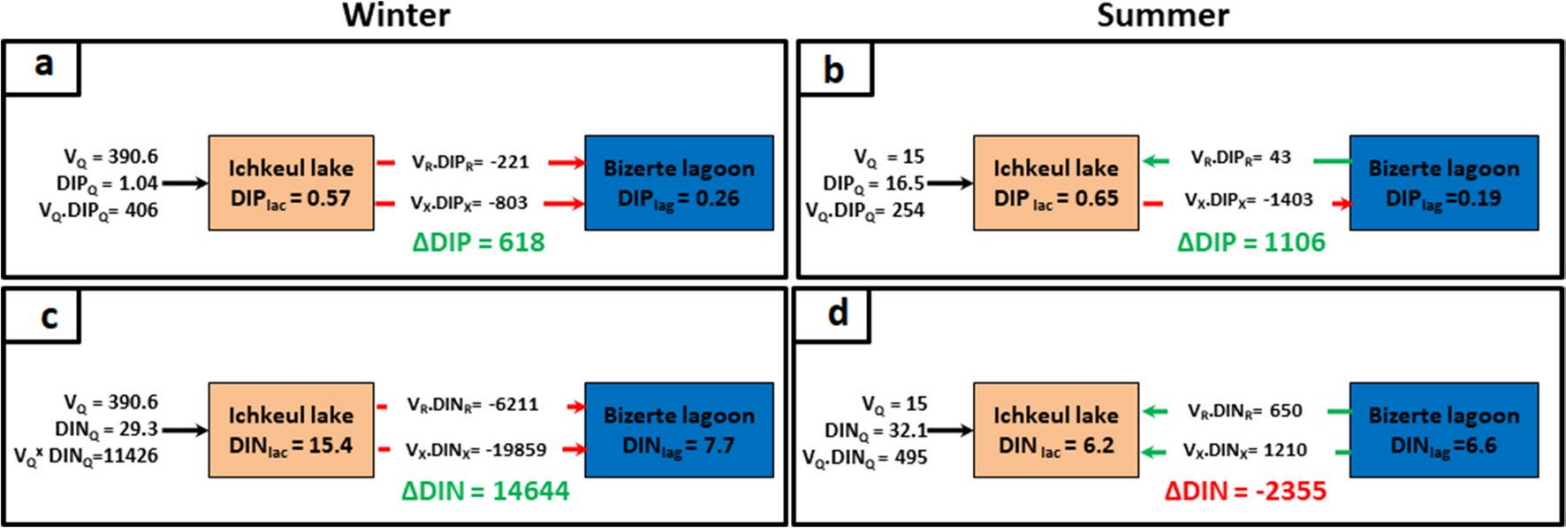
Generalized box diagrams illustrating DIP and DIN budgets in winter (**a**, **b**) and summer (**c**, **d**) respectively.

To estimate the DIP budget, the different fluxes of DIP were considered, namely, the rivers flux, the residual flux and the mixing flux. Then, accordioning to "Eq. (12)", during the winter season, the ∆DIP was about 618 mol P d^−1^ (Fig. 5a) while in summer it was about 1,106 mol P d^−1^ (Fig. 5b), indicating that for winter and summer, the Ichkeul Lake acted as a source of DIP.

##### DIN budget

The same procedure was adopted to estimate the DIN, therefore different fluxes were considered. Hence, in winter, the ∆DIN was positive at about 14,644.0 mol N d^−1^ (Fig. 5c), indicating that the lake acted as a source of DIN while in summer it was about − 2,355 mol N d^−1^ (Fig. 5d), indicating that the lake acted as sink of DIN.

To estimate the amount of dissolved nitrogen, not assimilated by phytoplankton and seagrass, the observed and expected DIN was performed under the Redfield approximation. Thereby, in winter, the expected **∆**DIN was about 9,888.0 mol N d^−1^ for phytoplankton and 18,540.0 mol N d^−1^ for seagrass. Therefore, the difference between N-fixation and denitrification was 4,756.0 mol N d^−1^ and − 3,896.0 mmol N d^−1^ for phytoplankton and seagrass, respectively. In summer, the expected ∆DIN was 17,696.0 mol N d^−1^ for phytoplankton and 33,180.0 mol N d^−1^ for seagrass, indicating that N-fixation minus denitrification was − 20,051.0 mmol N d^−1^ and − 35,535.0 mmol N d^−1^ for phytoplankton and seagrass, respectively.

#### Net Ecosystem Metabolism

In the LOICZ budget model, the value of Net Ecosystem Metabolism (NEM) is an indicator of the trophic metabolism; being the difference between contributions of primary production and respiration processes. In winter, the NEM was about − 65,508.0 and − 339,900.0 mol C d^−1^ for phytoplankton and seagrass, respectively; whereas in summer, the NEM was about −117,236.0 for phytoplankton and − 608,300.0 mol C d^−1^ for seagrass (Table 3). In the Ichkeul Lake, the NEM values were negative in both seasons, thus indicating that, under the present conditions, the respiration prevails over the production; suggesting that the Ichkeul Lake could be considered as heterotrophic ecosystem under high environmental threats.

**Table 3:** Residual (*V*_*R*_), the mixing flux (*V*_*X*_), the internal nitrogen fluxes namely the residual amount of Nitrogen-fixation and denitrification (*Nfix-Denit*) and the Net Ecosystem Metabolism (*NEM*) from numerical experiments.

	V_R_ (10^3^ m^3^ d^−1^)	V_X_ (10^3^ m^3^ d^−1^)	Nfix-Denit (mol N d^−1^)		NEM (mol C d^−1^)	
			Phytolankton	Seagrass	Pytolankton	Seagrass
Wet season	− 538.2	2,589.5	4,756.0	− 3896.0	− 65,508.0	− 339,900.0
Dry season	101.4	3,097.2	− 20,051.0	− 35,535.0	− 117,236.0	− 608,300.0

## Discussion

The Ichkeul Lake, at the heart of the coastal wetland ecosystem and as a shallow freshwater body, has also been shown to be seriously impacted by the damming upstream and the control of water exchange with the adjacent Lagoon and sea via the construction of locks which led to decreasing water levels, fluxes, and high salinity. Such mismanagement of water input and sediment deposits compounded by climate change would add further multiple stressors to the lake and associated ecosystems in the future. Indeed, it has been recently reported that climate change may increase the vulnerability of wetlands in the Mediterranean. The cumulative evaporation in the region far exceeds the cumulative precipitation leading to negative water balances that are aggravated by mismanagement of water resources and thereby has led to further water scarcity in one of the most affected Ecoregions.

The calculation performed by the LOICZ model regarding the residual volume flux in winter (− 538.2 10^3^ m^3^ d^−1^) and in summer (+ 101.4 10^3^ m^3^ d^−1^), leading to the residual flux in winter (− 18,406.4 kg d^−1^) and in summer (+ 3,978.9 kg d^−1^) depicts the high salinity observed in the Lake reaching 40. Notably, with a residence time of 10 days and inconsiderable river input, in respect to water fluxes exchanges with the lagoon, the lake behaves as a part of the lagoon, with hypersaline properties. On the contrary, in winter the lake behaves like a standard lake.

Human activities have affected not only the hydrological conditions, but also the water quality of the lake. For instance, the Joumine catchment area, a sub basin of Ichkeul watershed, is marked by important landforms, admitting surface runoff and nitrate leaching towards the Joumine river and then towards the lake^68^. Meanwhile, the region is surrounded by rural settlements with no sewage treatment systems. Swages are freely discharged, either in septic tanks or directly into the water circulation routes, leading to a risk of surface water contamination of the Lake^69^.

Moreover high loads of nitrate and phosphate are coming from important agricultural activities (especially cereals and sunflowers) and delivered to the Ichkeul Lake through the important hydrographic network surrounding the lake^76^. The western sector of the lake, which bears the highest nutrient concentration in winter, is surrounded by several land based pollution sources such as cereal crops, rural settling not connected to a sewage system, important cattle breeding areas and various economic activities such as the oil refinery, the Surgery Steel Company close to Menzel Bourguiba, and the Mineral Water firm. We also assume that the high concentration of phosphorus and nitrogen observed in the northern sector close to the irrigated agricultural fields is linked to the utilization of large quantities of chemical fertilizers in the agricultural land, and not to neglect urban discharges^67,68^.

These nutrient loads have accelerated the growth of vegetation biomass and the development of potentially toxic phytoplankton observed in the lake^70^.The high phytoplankton and vegetation biomass have impacted the dissolved oxygen to be close to saturation in link with photosynthetic activities^22^.

In relation to the phytoplankton growth limiting factors, the calculation of the DIN/DIP ratio shows that phytoplankton growth is essentially limited by phosphorus. Except for few sectors located on the northern shore of the lake, the phosphorus is quite available owing to intense agricultural activities. It is anticipated that phytoplankton is P-limited in Ichkeul Lake due to the shallow depth^78^. Studies on other lagoons in northern Tunisia have shown that phytoplankton proliferation depends on nitrogen: case of the Bizerte Lagoon^17^ and Tunis lagoon^79^. Based on our dataset, the DIN/DIP ratio is between 11 and 55 in winter and 5 and 60 in summer affirming an imbalance in the functioning of the lake and supports the hypothesis of the release of nitrogen into the atmosphere in the form of Nitrous gas.

The TRIX index^17^ has revealed very poor water quality (5.61 < TRIX < 8.46). The residence time reaching 1 month (31 days in winter) may contribute to the development of phytoplankton and macroalgae blooms and generating episodes of eutrophication in the lake. The Ichkeul Lake being a Biosphere Reserve and a RAMSAR Site, biomanipulation measures to control eutrophication are not permitted. Therefore, the best procedures to control localized anoxia and eutrophication would be an improved management of the water fluxes and the organic waste from towns and cities bordering the Lake^72^. Indeed, several lakes and water bodies that have undergone an increase in eutrophication and anoxia have witnessed a drastic rise and increase in phytoplankton and macroalgal blooms with the recent development in climate change effects. In many cases, the blooming species were toxic to food webs and humans, and could represent a real threat to the large diversity of the waterfowls of the Lake Ichkeul under the projected climate change scenarios for the Mediterranean region^73–75^.

To highlight the quantitative relationship between the different parameters, a multivariate statistical analysis was carried out. Thereby, the multivariate analysis suggests that the trophic status in the lake is phosphorous-dependent in winter and nitrogen-dependent in summer. In winter, the gradients between the areas close to the inlet and the coastal area near the mouth of the Douimis River, captured by the first two principal components of the PCA, suggest that the hydrodynamic pattern driven by the influence of the Lagoon from one side and the effect of Douimis River from the other has a major influence in shaping the Lake biogeochemical functioning. In fact, the detected values of the dissolved oxygen in the area around the inlet were high (10 mg/l). They are, likely, resulting from the hydrodynamic effect between the Lake and the Lagoon. Similar results were observed for the Phosphorous (1.3 µM) and Chlorophyll *a* (15 µg/l) which showed that the highest values were around the mouth of the Douimis River. In general, in this season, the ordination of the station indicates a high heterogeneity among all stations and variables, with a less clear a posteriori geographical interpretation of results, relative to summer. Salinity has a more important ordination role in summer, possibly as a result of the rivers-lagoon gradients, and the pattern of nitrogen and phosphorus shows some dissimilarity, possibly related to the presence of different sources. The temperature gradient also differed from that of summer. In summer, the lake functioning was characterized by the superposition of a major gradient between the inlet and the Douimis River tracked by the trophic status variables Chlorophyll *a* and phosphorous; and by the dissolved oxygen and might possibly be explained by the hydrodynamic functioning. In the summer season, the lake functioning is driven by the influence of the inlets from one part and the Melah, Joumine and Tine Rivers from the other.

To understand the nutrients dynamic which directly affect the water quality and phytoplankton and vegetation, the results of the LOICZ model were interesting. The ∆DIP values indicate that during both the wet and the dry seasons, the Ichkeul Lake acts as a source of DIP with relatively weak flux (∆DIP = 0.005 mmol m^−2^ d^−1^ in winter and 0.018 mmol m^−2^ d^−1^ in summer). The ∆DIN values, instead, have opposite signs in winter as compared to summer, thus indicating that the Lake acts as a source of DIN in winter (∆DIN = 0.12 mmol m^−2^ d^−1^) and as a sink in summer (∆DIN = -0.04 mmol m^−2^d^−1^).

The difference between the ∆DIN measured in the lake and the ∆DIN expected by the model is assumed to be the gaseous form of nitrogen in the lake as N_2_ gas. The Di-Nitrogen is supposed to be equal to the difference between the N-fixation and denitrification. The Nitrogen fixation and denitrification are likely to be important pathways for non-conservative nitrogen flux, in warm coastal ecosystems^43,80^; the nitrogen being balanced by means of denitrification and fixation processes.

In the winter season, the N-fixation minus denitrification is about 4,756.0 and − 3,896.0 mol N d^−1^ for phytoplankton and seagrass, respectively, suggesting that the N_2_ fixation prevails over the denitrification for phytoplankton; however, for seagrass the denitrification prevails over N-fixation. It seems that the winter conditions, where waters are well oxygenated, favors the fixation of N by phytoplankton but inhibits fixation of N by seagrass. This observation could be explained by the confined surface sediment which is the main reservoir of the seagrass. In summer season, the N-fixation minus the denitrification was − 20,051.0 mol N d^−1^ and − 35,535.0 mol N d^−1^ for phytoplankton and seagrass, respectively. In both cases, the denitrification processes prevail over the N-fixation due to the environmental conditions illustrated by the relatively low oxygen concentration, high water temperature, and organic matter accumulation. During this season, the Net Ecosystem Metabolism (NEM = Production minus Respiration) determined through the Redfield molar ratio between the phosphorus and the Carbon in the ecosystem is about − 65,508.0 (− 0.5) and − 339,900.0 (− 2.8) mol C d^−1^ (mmol C m^−2^ d^−1^) for phytoplankton and seagrass, respectively. These negative values suggest that respiration is prevailing in comparison to production and is attributed to the environmental conditions within the ecosystem. The ecosystem is therefore qualified as a confined ecosystem, whereas the metabolism is considered as heterotrophic.

In summer, unlike the wet season, the lake has shown different aspects. The difference between the observed and the expected ∆DIN may be attributed to the N_2_ amount generated by the denitrification and/or anammox, the process of ammonium and nitrite transformation into N_2_ gas by chemoautotrophic bacteria in anoxic conditions^81^. For Ichkeul Lake the Nfix-Denit is usually negative: at about − 20,051.0 (− 0.33) for phytoplankton and − 35,535.0 (− 0.58) mol N d^−1^ (mmol N m^−2^ d^−1^) for seagrass, respectively, indicating that the denitrification/anammox processes are dominant in the Lake. The NEM fluxes is about −117,236.0 (−1.9) and − 608,300.0 (-9.9) mol C d^−1^ (mmol C m^−2^ d^−1^) for phytoplankton and seagrass, respectively, indicating that respiration exceeds production and reinforcing the idea that a considerable amount of nitrogen gases are diffused to the atmosphere through the interface Atmosphere-Lake, either as N_2_ or as N_2_O.

The Ichkeul ecosystem showed a negative difference between production and respiration; and this difference illustrates that the lake responds to inputs of organic matter from surface runoffs by increasing respiration rates. Likewise, this indicates the dominance of heterotrophic metabolism, during both seasons, which is similar to the Lagoon of Venice, Sacca di Goro, and Stagnone di Marsala^28^. The dominance of this process, which is more obvious in summer (− 693.50 mmol C m^−2^ yr^−1^) than in winter (− 182.50 mmol C m^−2^ yr^−1^), may be explained by the warm water conditions that favor the bacterial activity and the oxidation of the organic matter^82^. On the other hand, other watersheds located in European regions revealed seasonal patterns of net metabolism, with the predominance of autotrophic metabolism in winter as compared to a dominance of heterotrophic metabolism in summer^28^.

The predominance of the nitrogen fixation process in winter, in Ichkeul Lake (14.60 mmol N m^−2^ yr^−1^) was similar to that found in Marinello-Verde (14.60 mmol N m^−2^ yr^−1^), but lower compared to Sacca di Goro (916 mmol N m^−2^ yr^−1^), Orbetello Lagoon (1347 mmol N m^−2^ yr^−1^) and S'Ena Arrubia (767 mmol N m^−2^ yr^−1^)^28^. The high N_2_ fixation in winter is a source of nitrogen that will be consumed in the system to boost the production^82^. Contrary to summer, the denitrification processes are mostly predominant^28^ and were also observed in various lagoons in Italy with seasonal denitrification patterns and subjected to high material loading during the warm season^82,83^. Similar results were reported for these lagoons as well as a strong correlation between denitrification and oxygen demand, and between denitrification and relatively high temperature rates. The predominance of denitrification is due to the environmental conditions prevailing in the ecosystem during the warm period. Indeed, the Ichkeul ecosystem is characterized by high temperatures (28.8 ± 0.6 °C) along with the lowest concentration of dissolved oxygen (6.8 ± 0.5 mg.l^−1^), and high inputs of nitrogen and phosphorus from agricultural activities and treated and untreated wastewater discharges^67,68,76^. The high nutrients load, which cannot be consumed by primary production due to poor climatic conditions, is released to the atmosphere in the form of N_2_ losses^31^. This highlights the role of coastal ecosystems in biological cycling at local, regional, and global scales, and their responses to excess nitrogen in addition to nutrient cycling^82^.

A recent study examining nitrogen processing and patterns in 34 lakes of the Midwest of the USA found that all lakes had a net loss of N_2_^84^. Comparable seasonal differences reported in our study have also been deduced in the Midwest lakes, with higher gaseous nitrogen escape forwarded by denitrification as related to anoxic conditions^85^. In addition, increased temperature from climate change, as it is the case for North-Africa, increases the production of N_2_ via the enhancement of the denitrification and/or anammox processes^86^. Most studies on the supersaturation of lakes in N_2_ have shown that nitrogen escapes to the atmosphere^28,30,31^. Yet eutrophication likely increases its escape to the atmosphere in its N_2_O form, thereby contributing to the greenhouse gas loading of the atmosphere; this is critical since N_2_O is 300 times more potent than CO_2_^87,88^. The recent effects of climate change and the development of human activities, notably the excessive use of agricultural fertilizers, warrant further research into the transfer of nitrogen to the atmosphere from wetlands and coastal wetland ecosystems.

The use of a Budget Model is important for coastal ecosystem because it provides the main features of the hydrological and biological functioning of the system and underlines certain unknown processes such as the N_2_ release to the atmosphere. The LOICZ budget approach provides a reliable estimation of the ecosystem’s functions (e.g. net heterotrophy and nutrient regeneration: or net autotrophy and nutrient retention) and permits to compare the system indicators with those calculated for other systems, thus providing a clear indication of the relative state of the system and of the restoration objectives^48^. In this context, a Budget Model provides a prior diagnosis before launching a heavy prognostic simulation which could require important computing resources. This approach depicts a preliminary picture of the main features, allowing the calibration and validation of numerical models.

## Methods

### Study area and main features

**Figure 6:**
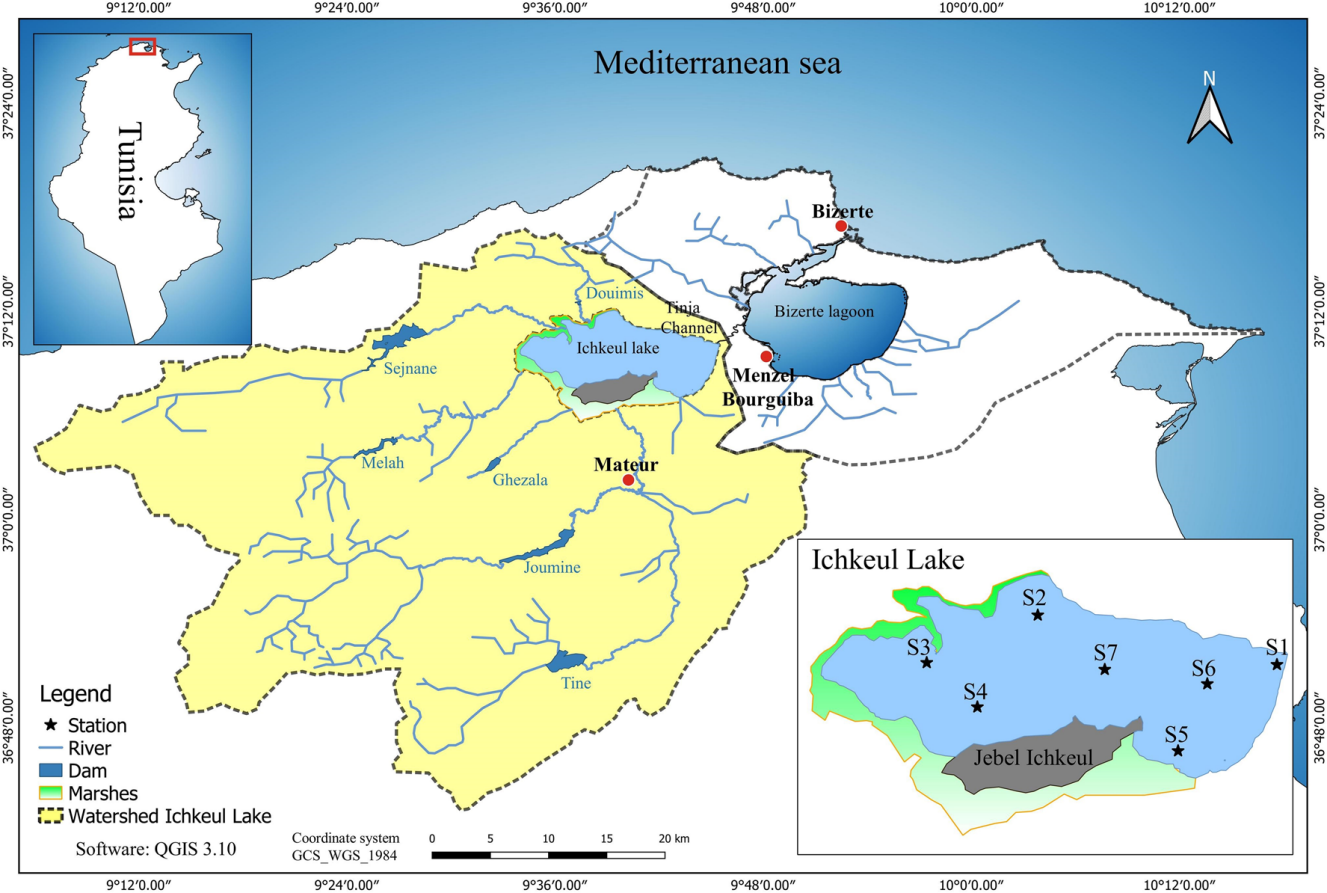
Geographic location of Ichkeul Lake and localization of sampling stations. Map created using QGIS 3.10 Software.

Ichkeul Lake, which is situated in the remote Northern Tunisia, is an ecosystem of 90 km^2^ surrounded by 30 km^2^ of temporary marshes^27^. The Southern shore of the lake is bordered by a limestone mountain—Jebel Ichkeul (Fig. 6). The Ichkeul Lake receives freshwater from a 2,600 km^2^ catchment area via six main rivers, and it is also linked by the Tinja channel to the coastal Bizerte Lagoon. The channel is about 5 km long and 3 m deep during the flood period ^90^, and it ensures the exchange of water and heat between the Ichkeul Lake and the Bizerte Lagoon.

This Lake is known for the seasonal fluctuations of water levels and salinity concentrations due to the variation of freshwater inputs between winter and summer, and it is characterized by its very shallow water depth, with a maximum depth of only 2–3 m during the wet seasons^20,22^. The water budget in the Ichkeul Lake depends on contributions from both marine waters from the Bizerte Lagoon and freshwater from direct rainfall and from its watersheds. During the rainy seasons, a large volume of the lake water spills off to the Bizerte lagoon through the Tinja channel^53^. In summer, high evaporation rates lower the water level and attract seawater towards the lake. The wetland is, therefore, characterized by a double seasonal alternation of high-water levels (2.0 m <) and low salinity (< 8.0) in the wet season from October to March due to freshwater from run-off, while it shows a low water levels (< 1 m) and a high salinity (50 <) in the dry season from April to September due to the inflow of sea water^27,53,91^.

The Ichkeul region has a Mediterranean climate with mild rainy winters and hot dry summers. The average annual rainfall is about 575.5 mm/year of which only 4% fall in the summer period. The mean temperature is about 19 °C and the potential evaporation is between 1,300 and 1,400 mm/year^92^.

### Experimental observations and statistical assessment

Water sampling at 7 stations (from S1 to S7, Fig. 6), in the Ichkeul Lake, was performed during 5 campaigns (November 2016, March, April, May, and August 2017). The locations of the sampling stations were selected according to the environmental and ecological conditions of the Lake.

In-situ measurements of water temperature (T), dissolved oxygen (DO), and salinity (S) were carried out by a pre-calibrated multi-parameter Thermo Orion meter. The laboratory analyses of the surface water samples were performed for the ammonium (NH4^+^), nitrates (NO_3_^-^), nitrites (NO_2_^-^) and phosphorous (PO_4_^3-^) using a BRAN and LUEBBE Auto-Analyzer-3, and their concentrations were determined calorimetrically using a UV–visible (6400/6405) spectrophotometer^100^. Total nitrogen (TN) and total phosphorus (TP) were determined after mineralization into ammonia and orthophosphate, respectively^93^. The Chlorophyll *a* (Chl*a*) concentrations were measured using the spectrophotometric method of Lorenzen (1967)^94^, following the procedure given by Parsons et al. (1984)^95^, after 24-h extractions in 90% acetone at 5 °C in the dark. The dissolved inorganic nitrogen (DIN) and the dissolved inorganic phosphorus (DIP) were computed for further analysis.

#### Multivariate statistical tool

A Principal Component Analysis (PCA) using R package Ade4 was performed to relate the sampling stations, Chl*a*, physico-chemical and chemical parameters in wet and dry seasons.

#### Material budgets model

**Figure 7:**
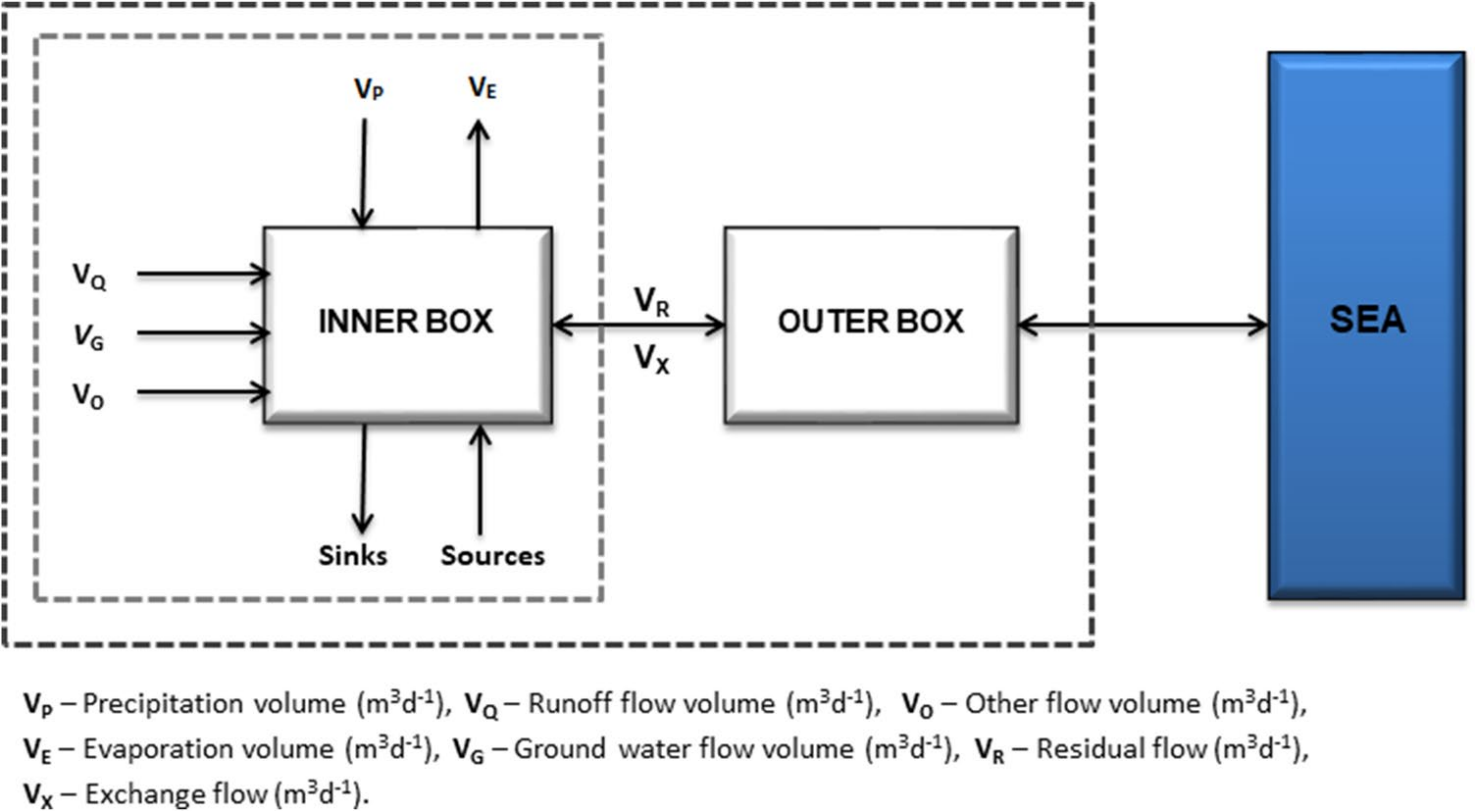
Land–Ocean Interaction in the Coastal Zone (LOICZ) budgeting procedure for the Ichkeul Lake. V_P_: Precipitation volume (m^3^d^−1^), V_Q_: runoff flow volume (m^3^d^−1^), V_0_: Other flow volume (m^3^d^−1^), V_E_: evaporation volume (m^3^d^−1^), V_G_: Groundwater flow volume (m^3^d^−1^), V_R_: residual flow (m^3^d^−1^), V_X_: Exchange flow (m^3^d^−1^).

Budget models are used to assess the budgets of physical and biological variables in coastal ecosystems. The Land Ocean Interaction Coastal Zone (LOICZ) model approach was used to assess water, salt, and non-conservative material budgets in the Lake. This tool has been proven to efficiently describe essential features of lagoon systems in the Mediterranean context^28,41^. The LOICZ was applied to scrutinize the impact of Climate Change on the coastal ecosystem and also to evaluate the strain of the anthropological effect^40^. The system intertwining Ichkeul Lake-Bizerte Lagoon is reproduced by the model as a simple single-layer box, simulating the Ichkeul Lake input–output fluxes on the entry/exit sides of the box, respectively (Fig. 7).

##### Water budget

The water volume of the Lake, *V*_*sys*_, is based on the mass balance equation "Eq. (1)" representing the water storage in the lake. In the steady state, the water budget equation allows the computation of the residual flux (*V*_*R*_) "Eq. (3)" which represents the amount of freshwater exchanged between the Ichkeul Lake and the Bizerte Lagoon. The theoretical flux (*V*_*R*_) is foreseen as the flux which restores the equilibrium of the Lake at the steady-state condition and could be either an outflow or an inflow. The horizontal water exchange fluxes *V*_*in*_ and *V*_*out*_ are hydrographically depicted by advection entering and leaving the system, respectively. Their difference is the residual flux.

The fluxes to the system are under several forms which are inflows from the rivers (*V*_*Q*_), direct precipitation (*V*_*P*_), ground water (*V*_*G*_) and other sources (*V*_*O*_) not streaming on the ground such as sewage and industrial waste. The out fluxes with respect to the Ichkeul Lake are under the form of evaporation (*V*_*E*_). In the LOICZ, budget computation approach is based on the consideration of steady-state (*V*_*sys*_ and V_R_ are constants). In the present study, the groundwater volume (*V*_*G*_) and other volumes (*V*_*O*_) such as industrial and sewage waste are ignored. The difference between the inputs and outputs to and from the lake are called *V*_Q*_and is expressed by "Eq. (2)".

1 $$\frac{{dV_{lac} }}{dt} = V_{in} - V_{out} + V_{{Q^{*} }}$$

2 $$V_{{Q^{*} }} = V_{Q} + V_{P} + V_{G} + V_{O} - V_{E}$$

3 $$V_{R} = V_{in} - V_{out} = \frac{{dV_{lac} }}{dt} - V_{{Q^{*} }}$$

In the steady state condition:

4 $$V_{R} = - V_{{Q^{*} }} = V_{E} - (V_{P} + V_{G} + V_{O} + V_{Q} )$$

##### Salt budget

The salt budget is estimated based on the amount of seawater exchanged between the system and the ocean due to the influence of currents exchanging materials (*mixing flux V*_*X*_). This flux does not lead to significant changes in volume and is, therefore, not included in the water budget. However, it is very important for nutrient flows as a mass of nutrient-rich system water is replaced by an equal mass of nutrient-poor seawater. To quantify *V*_*X*_, the salt budget which has a conservative behavior can be used to estimate the mixing flux. Therefore, the salt flux not considered by the salinities used to describe the freshwater, should be balanced by the mixing flux Vx*(S_lag_-S_lac_); although in freshwater systems, the mixing flux tends to be null ^43^.

At steady state, the salt mixing flux (*V*_*X*_) is equal to the sum of the residual flux which is carrying an amount of salinity (*S*_*R*_) described as the salinity of the residual flow "Eq. (7)", and the freshwater inflow flux which is carrying an amount of salinity (*S*_Q_*) "Eq. (9)". The quantity of water exchanged through the residual and the mixing flux allows for the estimation of the residence time of materials within the system "Eq. (10)".

5 $$\frac{{d\left( {V_{lac} S_{lac} } \right)}}{dt} = V_{in} S_{lag} - V_{out} S_{lac} + V_{{Q^{*} }} S_{{Q^{*} }}$$

6 $$V_{R} S_{R} = V_{in} S_{lag} - V_{out} S_{lac}$$

7 $$S_{R} = \frac{{\left( {S_{lac + } S_{lag} } \right)}}{2}$$

8 $$\frac{{d\left( {V_{lac} S_{lac} } \right)}}{dt} = V_{R} S_{R} + V_{{Q^{*} }} S_{{Q^{*} }}$$

In the steady state condition:

- Mixing Flux is,
  9 $$V_{X} (S_{lag} - V_{lac} ) = - (V_{R} S_{R} + V_{{Q^{*} }} S_{{Q^{*} }} )$$
- Water renewal time is,
  10 $$\tau= \frac{{V_{lac} }}{{\left( {V_{X} + \left| {V_{R} } \right|} \right)}}$$

##### Non-Conservative variables budget

Non-conservative materials (Y: DIP, DIN) owe their names to their non-conservativeness with respect to water and salt and could be considered as reactive substances. They are called so since their exchange fluxes through the lake are expected to leave an internal residual flux (∆Y), due to the internal processes occurring within the lake. The budgets for non-conservative materials follow a salinity-based approach but consider the internal flux.

In the present work, the Dissolved Inorganic Nitrogen (DIN) is represented by the sum of nitrites NO_2_^-^, nitrates NO_3_^-^ and ammonia NH_4_^+^ while the Dissolved Inorganic Phosphorous (DIP) is represented by phosphate PO_4_^3-^. LOICZ estimates the non-conservative materials DIN and DIP budgets at steady state by determining the residual flux of DIP and DIN namely ∆DIP and ∆DIN, respectively "Eq. (12)".

11 $${\text{V*}}\frac{{{\text{dY}}}}{{{\text{dt}}}} + {\text{Y*}}\frac{{{\text{dV}}}}{{{\text{dt}}}} = \sum \left( {V_{in} {*}Y_{in} } \right) - \sum \left( {V_{out} {*}Y_{out} } \right) + \Delta {\text{Y}}$$

At steady state:

12 $$\Delta {\text{Y}} = - (V_{{Q^{*} }} Y_{{Q^{*} }} + V_{R} Y_{R} + V_{X} \left( {Y_{lac} - Y_{lag} } \right)$$

The value of *Y*_*R*_ computed as the average between *Y*_*lac*_ and *Y*_*lag*_ is given by "Eq. (13)".

13 $$Y_{R} = \frac{{\left( {Y_{lac} + Y_{lag} } \right)}}{2}$$

##### Stoichiometric coefficients

Mass-balance budgets are described to stoichiometrically link nutrient budgets through C:N:P Redfield ratios^96^. LOICZ principally uses those ratios to determine the excepted *∆DIN*, *Nfix-Denit* and Net Ecosystem Metabolism (*NEM*). The expected ∆DIN could be estimated through the observed ∆DIP^97^. The difference between the observed and expected ∆DIN "Eq. (14)" is supposed to be equal to the difference of the quantity of N_2_ produced through denitrification and that is consumed through fixation^98^. As for the NEM, it is supposed to reflect the difference between the *production* and *respiration* (p-r) within the lake, estimated (in Carbon) through the observed ∆DIP, exploring the Redfield ratio. The P:C Redfield ratio is about (106:1) for phytoplankton^96^ and (550:1) for sea grass^99^.

14 $$Nfix - Denit = \Delta \left( {DIN} \right){-}\Delta \left( {DIN} \right)_{exp}$$

15 $$NEM = p - r = - \Delta \left( {DIP} \right)*\left( {C:P} \right)$$

## Supplementary Information

Supplementary Figure 1.

Supplementary Legends.
