# Supplementary figures and images for: Hydrology, biogeochemistry and metabolism in a semi-arid mediterranean coastal wetland ecosystem
Source: Sci Rep. 2022 Jun 7;12:9367. doi: 10.1038/s41598-022-12936-5 (PMC9174276; doi:10.1038/s41598-022-12936-5)

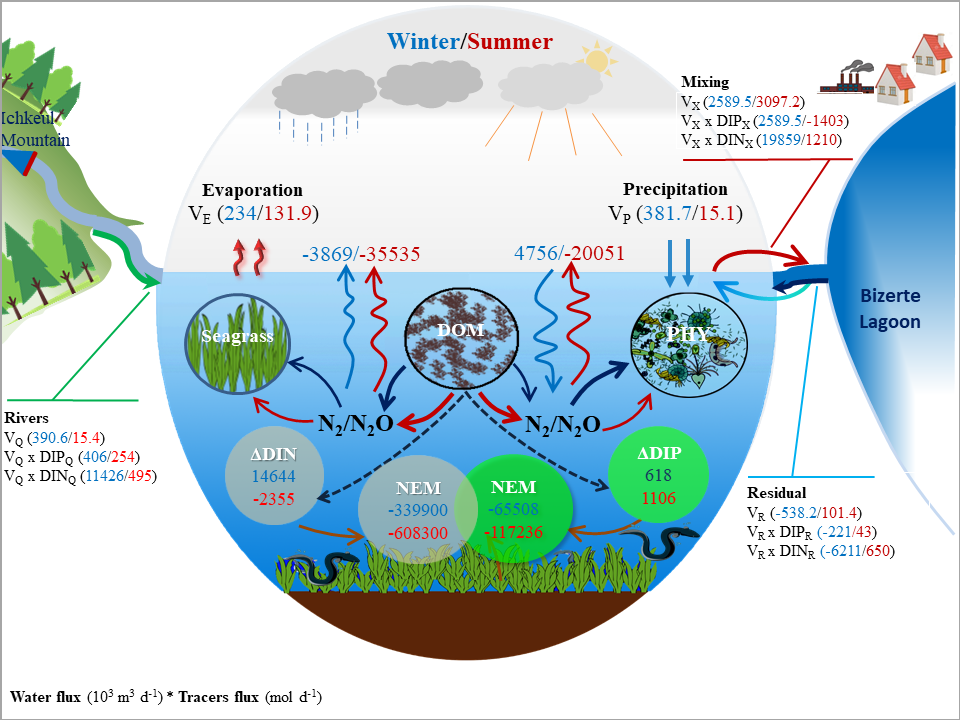

Supplement: Supplementary file 1 — Supplementary Figure 1. [file 41598_2022_12936_MOESM1_ESM.png]
