# Supplementary material for: Hydrology, biogeochemistry and metabolism in a semi-arid mediterranean coastal wetland ecosystem
Source: Sci Rep. 2022 Jun 7;12:9367. doi: 10.1038/s41598-022-12936-5 (PMC9174276; doi:10.1038/s41598-022-12936-5)
Supplement: Supplementary file 2 — Supplementary Legends. [file 41598_2022_12936_MOESM2_ESM.docx]

**Figure SM1.** Summary of the seasonal hydrological, biogeochemical and metabolic functioning of the Ichkeul Lake wetland ecosystem
